# Novel Inhibitors and Activity-Based Probes Targeting Trypsin-Like Serine Proteases

**DOI:** 10.3389/fchem.2022.782608

**Published:** 2022-04-21

**Authors:** Timothy E. G. Ferguson, James A. Reihill, S. Lorraine Martin, Brian Walker

**Affiliations:** Biomolecular Sciences Research Group, School of Pharmacy, Queen’s University Belfast, Belfast, United Kingdom

**Keywords:** trypsin-like proteases, inhibitors, activity probes, activity-based profiling, trypsin, cystic fibrosis, cockroach extract

## Abstract

The trypsin-like proteases (TLPs) play widespread and diverse roles, in a host of physiological and pathological processes including clot dissolution, extracellular matrix remodelling, infection, angiogenesis, wound healing and tumour invasion/metastasis. Moreover, these enzymes are involved in the disruption of normal lung function in a range of respiratory diseases including allergic asthma where several allergenic proteases have been identified. Here, we report the synthesis of a series of peptide derivatives containing an *N*-alkyl glycine analogue of arginine, bearing differing electrophilic leaving groups (carbamate and triazole urea), and demonstrate their function as potent, irreversible inhibitors of trypsin and TLPs, to include activities from cockroach extract. As such, these inhibitors are suitable for use as activity probes (APs) in activity-based profiling (ABP) applications.

## Introduction

The trypsin-like proteases (TLPs) catalyse the hydrolysis of peptides and proteins at arginine and lysine residues and are one of the most widely studied group of enzymes in biology. Interest in these hydrolytic enzymes is due to their well-characterized, widespread, and diverse roles, in a host of physiological and pathological processes. For example, it has long been established, that in addition to their fibrinolytic role in clot dissolution (Yaron et al., 2021), the trypsin-like serine proteinases urokinase (uPA), tissue-type plasminogen activator (tPA) and plasmin play critical roles in a number of processes including extracellular matrix remodelling (Lu et al., 2011), wound healing and carcinogenesis (Aimes et al., 2003; Nyberg et al., 2006; Pawar et al., 2019).

The most comprehensively studied groups of TLPs are those involved in the coagulation cascade (thrombin, protein C, factors VIIa, IXa, Xa and XIIa) and complement system (C1r, C1s, C3 convertase, C5 convertase and factor D) (Forneris et al., 2012; Oncul and Afshar-Kharghan, 2020; Winter et al., 2020). Classical biochemical studies carried out on the clotting factor proteases (such as those alluded to above) have been foundational to the development of a number of novel orally active anticoagulants that function as direct inhibitors of thrombin and factor Xa (Liang and Bowen, 2016). For example, Apixaban and Rivaroxaban (both of which inhibit factor Xa) and Dabigatran (a thrombin inhibitor), are now routinely used for the prevention of thromboembolic events that can occur after orthopaedic surgery to replace hip and knee joints (Samama, 2011; Samama et al., 2020; Hasan et al., 2021). These drugs have also been approved for use as prophylactics in reducing venous and arterial thrombosis, and for stroke prevention in patients with atrial fibrillation (Harter et al., 2015; Schulman et al., 2017; Chen et al., 2020; Arora et al., 2021; Bulwa et al., 2021).

Other TLPs such as tryptase, matriptase, prostasin, human airway trypsin-like protease (HAT) and TMPRSS2 have been implicated in respiratory diseases such as cystic fibrosis (CF), chronic obstructive pulmonary disease (COPD) and asthma (Akers et al., 2000; Bardou et al., 2016; Menou et al., 2017; Reihill et al., 2020; Carroll et al., 2021), as well as COVID-19 where TLPs are involved in the activation of the SARS-CoV-2 spike protein allowing cellular entry (Hoffmann et al., 2020). TLPs have also been identified as facilitators of parasite infectivity (Horn et al., 2014; Yang et al., 2015; Leontovyc et al., 2018) and some allergen TLPs have potent effects that severely impact human health (Sudha et al., 2008; Arizmendi et al., 2011; Polley et al., 2017; Reithofer and Jahn-Schmid, 2017; Reihill et al., 2020). It is therefore important that detection methodologies are developed that can lead to the disclosure and identification of members of this protease family. Opportunities to pinpoint their precise roles across this myriad of biological, physiological and pathological processes could help highlight important targets for future drug development.

This paper reports on the synthesis of a series of peptide derivatives containing an *N*-alkyl glycine analogue of arginine, bearing differing electrophilic leaving groups (carbamate and triazole urea), and demonstrates that they function as potent, irreversible inhibitors not just of trypsin but a range of TLPs, making them suitable for use as activity probes (APs) in activity-based profiling (ABP) applications.

## Materials and Methods

### Materials

All reagents and solvents were from Sigma-Aldrich unless otherwise indicated. All standard Fmoc-protected amino acids were supplied by Activotec. Fmoc-NArg (Pbf)-OH was supplied by PolyPeptide Laboratories. Biotin-PEG NovaTag resin was supplied by Merck Millipore and Fmoc-Lys (Mtt)-Wang resin was supplied by Bachem. ESI-MS analysis was carried out by ASEPT (Queen’s University Belfast). HPLC analysis of final compounds was carried out on an Agilent Technologies 1,260 Infinity machine using a Waters Atlantis C18 5 μm, 4.6 × 150 m column. A two-phase solvent system was used, A) 0.05% (v/v) TFA in water and B) 0.05% (v/v) TFA in acetonitrile. A linear gradient elution system was implemented at 1 ml/min from 0% B) to 90% B) over 45 min, held for a further 10 min, then back to 0% B) over 10 min and held for a final 10 min. A UV detector was used to monitor absorbance at λ = 216 nm. Prostasin, matriptase, Human Airways Trypsin-like protease (HAT) and thrombin, were supplied by R and D Systems. Human neutrophil elastase was supplied by Calbiochem. SDS-PAGE was carried out using NuPAGE Novex 4–12% Bis-Tris protein gels 1.0 mm using a PowerEase 500 power supply with a SeeBlue Plus2 pre-stained Protein Standard as a reference ladder, all supplied by Invitrogen. Rhodamine-X azide and Copper (II)-TBTA complex were supplied by Lumiprobe. Western blotting was achieved with an X-Cell II Blot Module (Invitrogen) unto an Amersham Hybond ECL Nitrocellulose membrane (GE Healthcare). Luminata Forte Western HRP substrate was supplied by EMD Millipore. Streptavidin-HRP was supplied by Vector Laboratories. Cockroach extracts were supplied from Greer Laboratories. Neutravidin agarose beads were supplied by Thermo Scientific.

### Synthesis of Inhibitors

**FIGURE 1:**
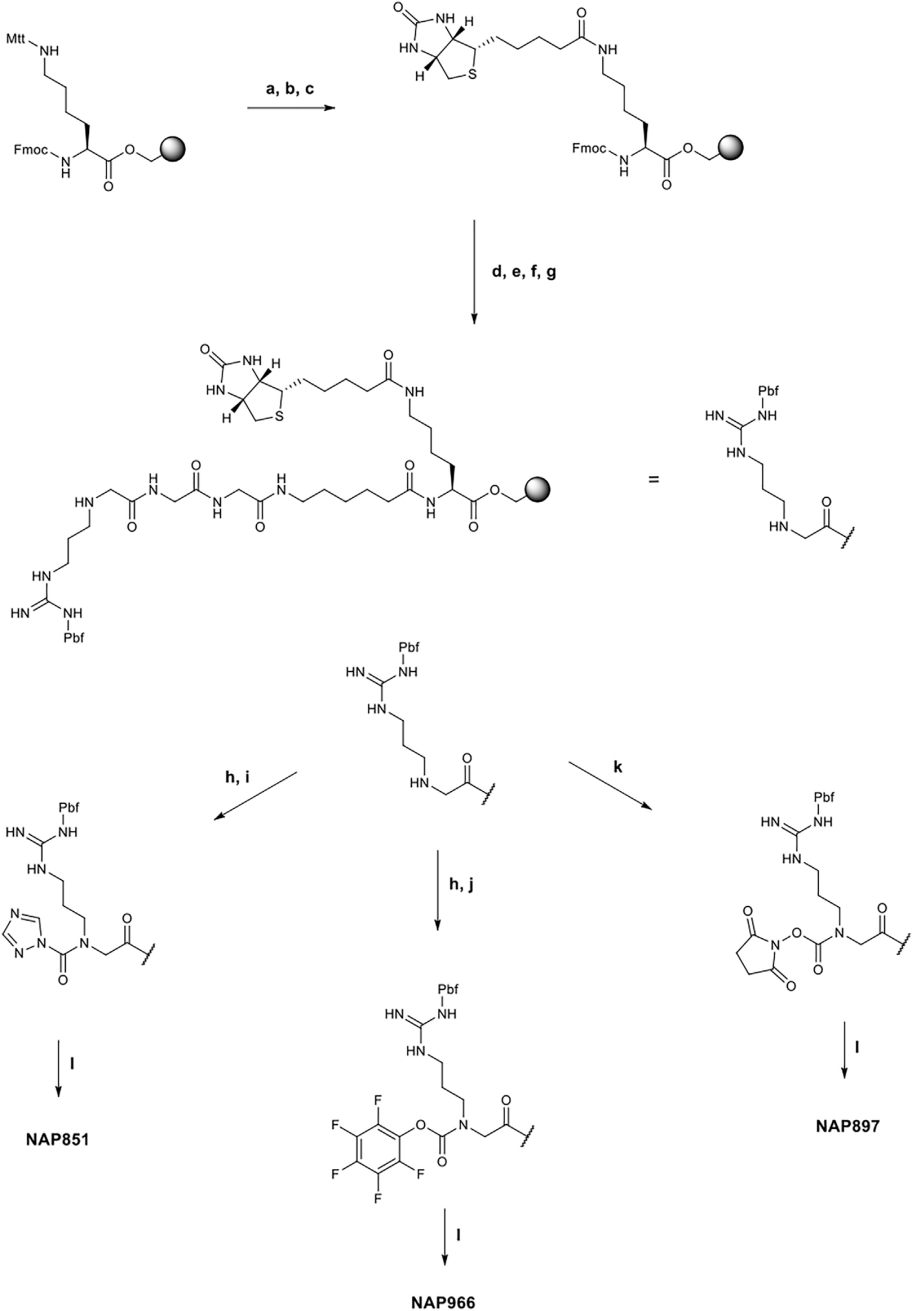
Synthetic schemes for the preparation of NAP851, NAP966 and NAP897. **(A)** TFA/TIPS/DCM (5/5/90, v/v/v), **(B)** DIPEA/DMF (1/99 v/v), **(C)** Biotin, DIC, HOBt, NMP, **(D)** i) Piperidine/DMF (1/5, v/v), ii) Fmoc-Ahx-OH, DIC, HOBt, DMF, **(E)** i) Piperidine/DMF (1/5, v/v), ii) Fmoc-Gly-OH, DIC, HOBt, DMF, then repeat i) and ii) for a second Gly residue, **(F)** i) Piperidine/DMF (1/5, v/v), ii) Fmoc-NArg (Pbf)-OH, DIC, HOBt, DMF, **(G)** Piperidine/DMF (1/5, v/v), **(H)** Triphosgene, 2,6-lutidine, anhydrous DCM, **(I)** 1,2,4-triazole, 2,6-lutidine, DMAP, anhydrous DCM, **(J)** Pentafluorophenol, 2,6-lutidine, DMAP, anhydrous DCM, **(K)** *N,N′*-disuccinimidyl carbonate, 2,6-lutidine, anhydrous DCM, **(L)** TFA/TIPS/DCM (95/2.5/2.5, v/v/v).

All compounds were synthesized using solid-phase peptide synthesis (SPPS) methods (full details of the synthetic methods are provided in the supplementary information). Briefly, the biotinylated compounds NAP851, NAP966 and NAP897 were synthesised on a Fmoc-Lys-(Mtt)-Wang resin. In each case, the synthesis was commenced by removal the Mtt group under mild acid conditions (TFA/TIPS/DCM: 5/5/90) and following neutralisation (DIPEA/DMF: 1:99), Biotin was then coupled to the newly unmasked ε-amino function of lysine (DIC/HOBt) and the resin-bound, common core peptide, Fmoc-NArg-(Pbf)-Ahx-Gly-Gly-Lys-(*N*-ε-Biotin)- was then constructed using standard Fmoc-SPPS methods (Walker, 1994), employing commercially available Fmoc-amino acids (Fmoc-Ahx-OH and Fmoc-Gly-OH) and the *N*-alkyl glycine arginine derivative, Fmoc-NArg (Pbf)-OH. Following the final Fmoc group removal to yield the free secondary amine (2^o^) of the NArg residue, the resin was spilt and the incorporation of the respective electrophilic groups into the core recognition sequence to yield NAP851, NAP966 and NAP897 was carried out whilst the peptide was still attached to the resin, as shown in Figure 1. Formation of the triazole urea moiety in NAP851 was achieved using a similar approach to that reported previously (Adibekian et al., 2011). In brief, once the final Fmoc deprotection had been performed, the NArg peptide-resin was treated with triphosgene and 2,6-lutidine in anhydrous DCM for 15 min, until chloranil analysis indicated complete consumption of the secondary N^α^ amine. The resin was then washed briefly with further anhydrous DCM, followed by addition of 1,2,4-triazole, 2,6-lutidine and catalytic DMAP in anhydrous DMF, overnight, to allow formation of the triazole urea. The peptide was then simultaneously cleaved from the resin and the side-chain Pbf-protecting group removed using TFA/TIPS/DCM (95:2.5:2.5) and, following workup and precipitation in diethyl ether, the product was obtained in quantitative yield.

Incorporation of the *penta*-fluorophenyl carbamate into the common recognition sequence to yield NAP966, followed a similar method to that followed for the preparation of NAP851, with the exception that following treatment of the resin with triphosgene, a solution containing pentafluorophenol, 2,6-lutidine and catalytic DMAP in anhydrous DMF was added. Following cleavage and workup, as previous, the product was obtained in good yield.

The synthesis of the *N*-hydroxy-succinimide carbamate NAP897, was achieved in a much more straightforward method, according to the method of Niphakis *et al.* (Niphakis et al., 2013). Briefly, a solution containing *N,N′*-disuccinimidyl carbonate (DSC) and 2,6-lutidine in anhydrous DCM/DMF (1:1) was added to the deprotected resin until chloranil analysis indicated complete reaction of the secondary *N* ^*α*^ amine function. The peptide was cleaved as described for NAP851 and NAP966, giving the required compound in quantitative yield.

Compound NAP884, capable of undergoing ‘click chemistry’ reactions, was synthesised in an identical manner to that described for NAP897, with the exception that, following Mtt deprotection of the Fmoc-Lys-(*N*-ε-Mtt)-Wang resin, 4-(3-ethynyl-phenylcarbamoyl)-butyric acid, prepared according to a previously reported method (Sivakumar et al., 2004), was coupled to the ε-amino function instead of the previously used biotin reporter group. The rest of the inhibitor synthesis, formation of the NHS carbamate and cleavage was carried out as previously described to give the desired compound in reasonable yield.

**FIGURE 2:**
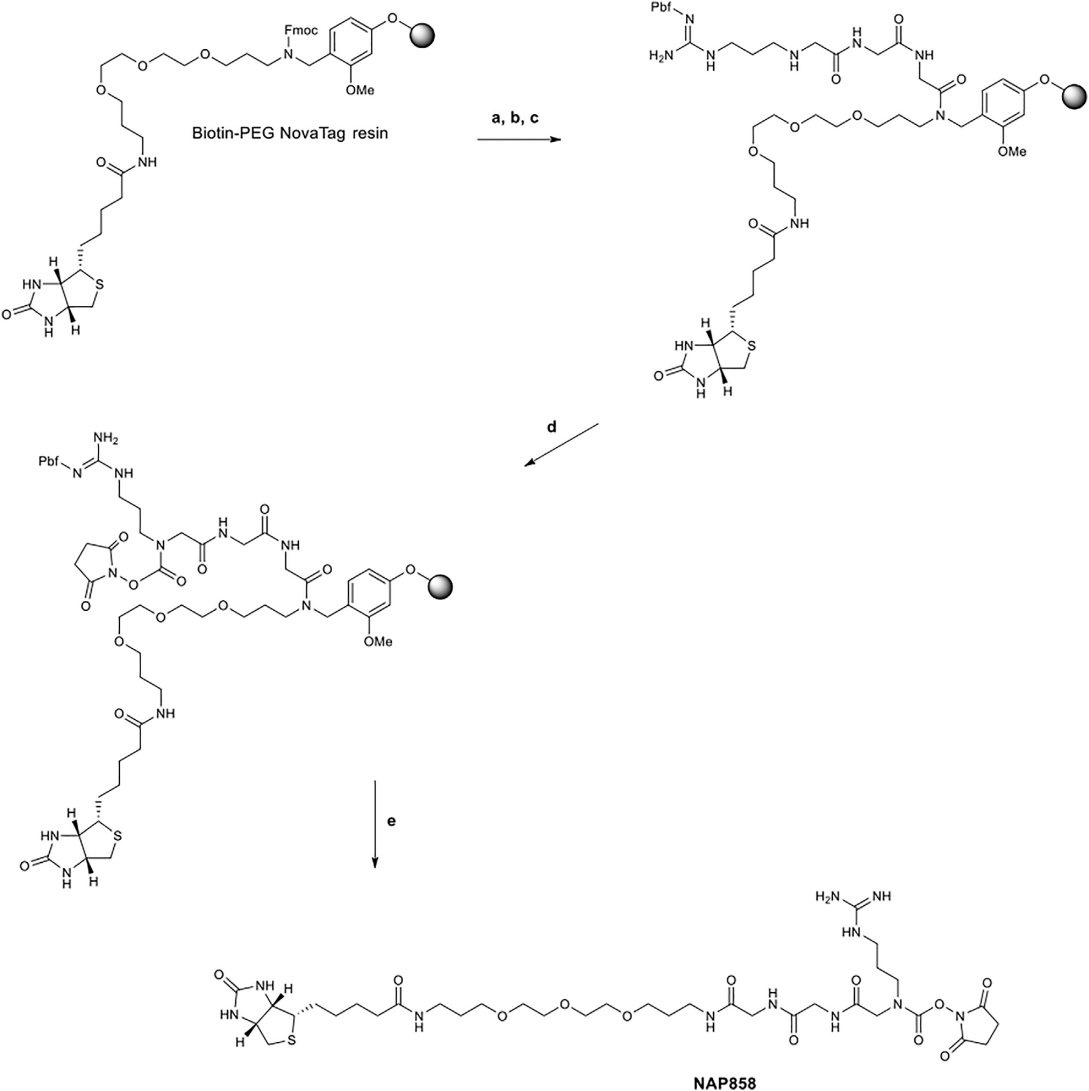
Synthetic scheme for the preparation of NAP858. **(A)** Piperidine/DMF (1/5, v/v), ii) Fmoc-Gly-OH, DIC, HOBt, DMF, then repeat i) and ii) for a second Gly residue, **(B)** i) Piperidine/DMF (1/5, v/v), ii) Fmoc-NArg (Pbf)-OH, DIC, HOBt, DMF, **(C)** Piperidine/DMF (1/5, v/v), **(D)** *N,N′*-disuccinimidyl carbonate, 2,6-lutidine, anhydrous DCM, **(E)** TFA/TIPS/DCM (95/2.5/2.5, v/v/v).

The synthetic scheme employed for the synthesis of the pegylated compound NAP858 is shown in Figure 2. Again, this target compound was prepared utilising exclusively Fmoc-SPPS methods, only in this instance; the synthesis was performed on Biotin-PEG NovaTag resin. This resin contains a pegylated-diamine residue functionalised with biotin at one amino function, with the other amino function coupled to the resin beads, through a benzyl linker in the form of an Fmoc-protected 2^o^ amine derivative. The synthesis of NAP858 was commenced by treating samples of the Biotin-PEG NovaTag resin with piperidine to remove the Fmoc-protecting group and the resin-bound 2^o^ amine was acylated with two consecutive couplings of Fmoc-Gly-OH, again using standard Fmoc-SPPS methods (Walker, 1994). The incorporation of Fmoc-NArg (Pbf)-OH into the target sequence and formation of *N*-hydroxysuccinimde (NHS) carbamate linkage was exactly as described above for the preparation of NAP897 and NAP884. The desired product was then simultaneously cleaved from the resin and the side-chain Pbf protecting group removed, using TFA/TIPS/DCM (95:2.5:2.5).

All synthesised inhibitors were analysed by MS and HPLC and gave satisfactory data consistent with their proposed structures (Table 1). ESI-MS Spectrum and HPLC chromatogram for NAP858 are shown in Supplementary Figure S1 and Supplementary Figure S2.

**TABLE 1:** Electrospray Ionisation Mass Spectrometry analysis of synthesised compounds.

Compound	Calculated monoisotopic Mass	Determined MW
*NAP851*	850.42	852.39 [M + H^+^]
*NAP966*	965.39	966.39 [M + H^+^]
*NAP897*	896.42	897.43 [M + H^+^]
*NAP858*	857.41	858.42 [M + H^+^]

### Kinetic Evaluation of Activity Based Probes

Recombinant protease activities were assessed using the cognate fluorogenic substrates (final concentration 50 μM) as follows: trypsin (Cbz-Gly-Gly-Arg-AMC), neutrophil elastase (MeO-Suc-Ala-Ala-Pro-Val-AMC) and chymotrypsin (Suc-Ala-Ala-Pro-Phe-AMC). The assay in the presence or absence of protease inhibitors at a range of final concentrations was performed using a standard microtitre plate format. Substrate hydrolysis, at 37°C, was monitored over a 60 min period by measuring the rate of increase in fluorescence (at λ_ex_ 360 nm, λ_em_ 480 nm) using a FLUOstar Optima microplate reader (BMG Labtech).

### Determination of Inhibitor Constants Against Trypsin

A broad range of inhibitor concentrations were prepared from a 10 mM stock solution of the five test compounds (NAP851, NAP966, NAP987, NAP884 and NAP858) in *N,N-*dimethyformamide (DMF). The fluorogenic substrate, Cbz-Gly-Gly-Arg-AMC, was diluted in assay buffer (PBS, pH 7.4) and used at a fixed concentration of 50 μM. All inhibition assays were performed in microtitre plates maintained at 37°C in a final volume of 100 μL. The reaction was initiated by the addition of human trypsin (0.01 μg/well) and the rate of substrate hydrolysis continuously recorded at λ_ex_ 360 nm, λ_em_ 480 nm using a FLUOstar Optima microplate reader (BMG Labtech). The resultant inhibition progress curve for the putative protease inhibitors were analysed according to the kinetic models developed by Tian and Tsou (Tian and Tsou, 1982) and Walker and Elmore (Walker and Elmore, 1984), for irreversible inhibitors, using GRAFIT (Erithacus Software) as described previously (Ferguson et al., 2016).

### Evaluation of Novel Protease Inhibitors as ABPs Using Recombinant Trypsin-Like Protease

Samples of recombinant protease (trypsin, human airway trypsin-like protease (HAT), matriptase, thrombin or neutrophil elastase, as indicated in the relevant figure legends) were treated with putative ABPs at a concentration of 50 μM, for 30 min, at 37°C. A control sample was prepared by treating recombinant protease with solvent control only. A further sample was prepared where Cbz-Arg^P^(OPh)_2_ was added 30 min prior to the addition of the ABP under examination. Pre-treatment of protease with a non-biotinylated, irreversible, active-site directed inhibitor, such as Cbz-Arg^P^(OPh)_2_ in the case of trypsin, should result in a quenching/diminution in the signal detected by the ABP, indicating that these probes are active-site directed and do not cause non-specific chemical modification of the protease. All treated samples were then denatured with SDS-containing reducing treatment buffer (10 min at 95°C), followed by resolution by SDS-PAGE and subjected to Western blotting. Note, only stable covalent bonds between the ABP and protease active site would survive the harsh denaturing conditions involved in the resolution process. Resolved proteins were then transferred on to nitrocellulose membrane and labelled with streptavidin-HRP. The ABP-protease complex was then visualised following treatment with Luminata Forte HRP substrate.

### Click Chemistry Labelling With NAP884

Samples of recombinant protease (trypsin and neutrophil elastase) were treated with alkyne-containing probe NAP884 at a concentration of 50 μM, for 30 min at 37°C. Samples pre-treated with competitive inhibitor (Cbz-Arg^P^(OPh)_2_ for trypsin and Cbz-Val^P^(OPh)_2_ for neutrophil elastase) for 30 min prior to addition of NAP884, were included for comparison. Following treatment with NAP884, DMSO (25% v/v final concentration), rhodamine-X azide (60 µM final concentration), copper (II)-TBTA complex (1 mM final concentration) and freshly prepared sodium ascorbate (1 mM final concentration) were added. The samples were mixed and incubated at room temperature in the dark for 1 h with gentle agitation. Samples were then denatured with SDS-containing reducing treatment buffer (10 min at 95°C), followed by resolution by SDS-PAGE and visualisation under UV light.

### Affinity Purification and Enrichment of Cockroach Extracts

Cockroach extract was probed with NAP858 (50 μM) (± heat inactivation at 95°C, for 10 min), for 30 min, at 37°C. Samples were then denatured with SDS-containing reducing treatment buffer (10 min/95°C), followed by resolution by SDS-PAGE and protein transfer unto nitrocellulose membrane which was incubated with streptavidin-HRP (1 in 20,000). The membrane was visualised following treatment with Luminata Forte HRP substrate.

Removal of endogenous biotinylated proteins present in the cockroach extract was performed in some experiments to improve clarity of the Western blots obtained. Neutravidin agarose beads were washed three times with 1 ml wash buffer (150 mM NaCl, 50 mM Tris, 1 mM EGTA, 1% (v/v) NP-40, pH 7.4) then resuspended in PBS (pH 7.4). These neutravidin beads were then used to bind and remove (pre-clear) endogenous biotinylated proteins present in the cockroach extract. This involved rotation of the bead-cockroach extract mix at 4°C for 30 min followed by centrifugation to enable collection of the pre-cleared supernatant fraction. The bead fraction containing bound endogenous biotinylated proteins from cockroach extract was discarded. The supernatant was then probed with NAP858 (50 μM) (±Cbz-Arg^P^(OPh)_2_), for 30 min, at 37°C, followed by addition of freshly washed neutravidin agarose beads. Following incubation (1 h/4°C), the beads containing bound TLPs were collected and washed with further wash buffer (x3). Bound proteins were eluted from the beads with SDS-containing reducing treatment buffer (10 min/95°C). Subsequent resolution by SDS-PAGE and transfer unto a nitrocellulose membrane, facilitated the detection of purified TLPs, following incubation with streptavidin-HRP (1 in 20,000) and Luminata Forte HRP substrate.

### Covalent Docking of NAP858

NAP858 was covalently docked to the side chain of SER195 of thrombin (PDB: 1DWE) as an example trypsin-like serine protease. The structure of NAP858 was converted to pdb format using UCSF Chimera 1.15 (Pettersen et al., 2004) and converted to PDBQT format by autodocktools 1.5.6 (Morris et al., 2009). The target file of thrombin (1DWE) was created using AutoGridFR 1.2 and the docking was performed using AutoDockFR 1.0 (Ravindranath et al., 2015). Final poses were reviewed, and images generated using UCSF Chimera 1.15.

## Results

**FIGURE 3:**
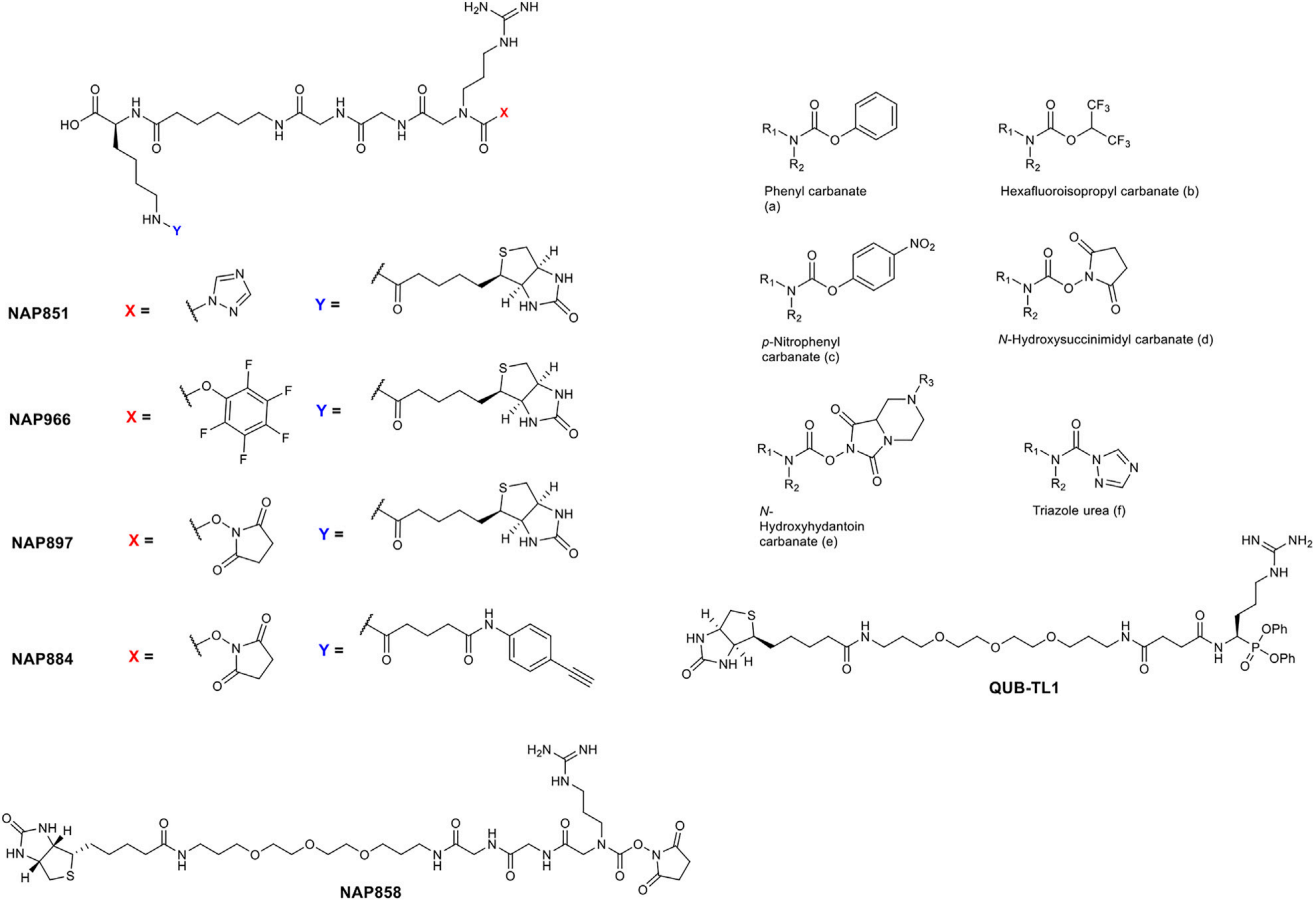
Comparison of the structures of arginine *N*-alkyl glycine triazole urea and carbamate analogues synthesised in the present study, with previously reported triazole urea and carbamate **(A–F)** inhibitors of the serine hydrolases. The structure of the arginine diphenyl-phosphonate analogue (QUB-TL1), a previously reported activity probe for TLPs (Reihill et al., 2016) is also shown.

**FIGURE 4:**
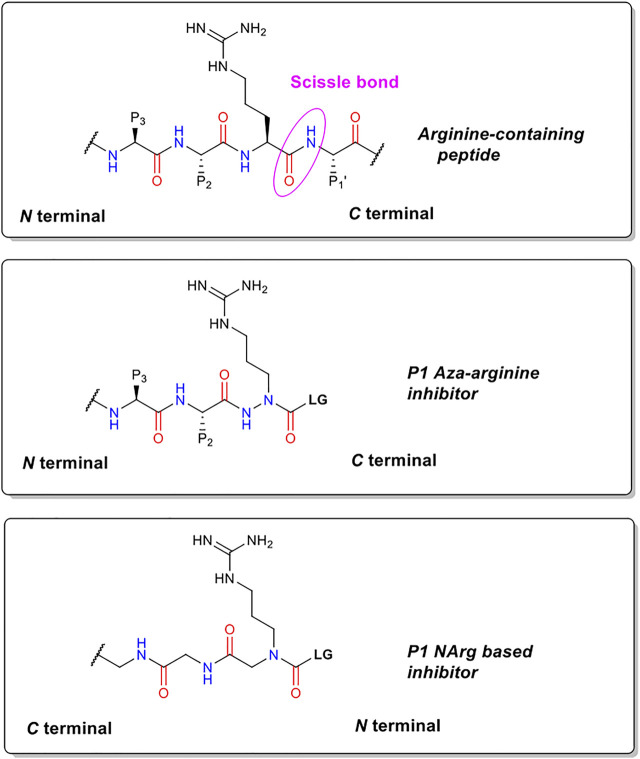
Structural comparisons of P1 NArg inhibitors with standard trypsin substrates and P1 aza-arginine containing inhibitors.

**FIGURE 5:**
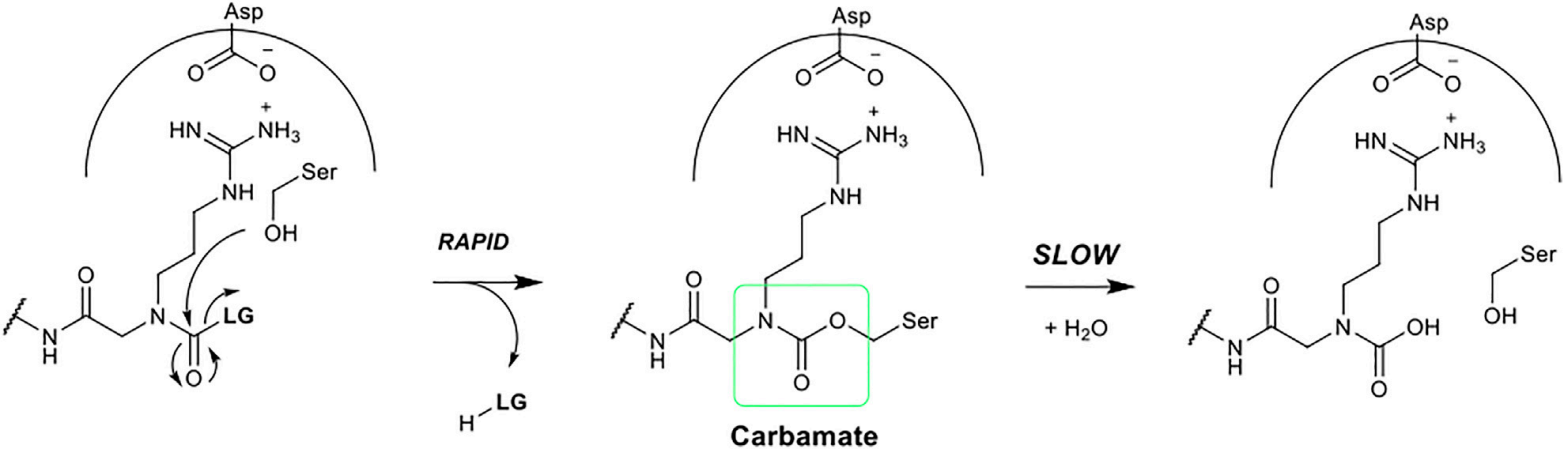
Postulated mechanism of serine protease inhibition by *N*-alkyl glycine inhibitors.

The precise chemical structure of each of the five compounds (NAP851, NAP966, NAP897, NAP884 and NAP858) synthesised in the present study is illustrated in Figure 3 and described also in US Patent No. 11, 104, 703 (issued 31st August 2021). For comparison, the structures of previously described triazole urea and carbamate (a-f) inhibitors of serine hydrolases (Adibekian et al., 2011; Chang et al., 2011; Chang et al., 2013; Cognetta et al., 2015) and an amino alkyl diphenyl-phosphonate arginine analogue QUB-TL1, previously developed by us, that functions as an AP for the TLPs are included (Reihill et al., 2016). We reasoned that these new compounds would bind to the active site of TLP due to their similarity in structure to aza-peptide protease inhibitors (Figure 4) (Chang et al., 2011; Chang et al., 2013). It was hypothesised that such compounds, containing a P1 NArg residue in combination with a good leaving group (LG), would result in a poorly hydrolysed, carbamate linkage between the inhibitor and the active site serine residue, effectively causing irreversible inhibition of the protease (Figure 5). This inhibition mechanism has been previously reported with a variety of carbamate-based inhibitors of serine hydrolases (Walker and Elmore, 1984; Sivakumar et al., 2004; Adibekian et al., 2011; Arizmendi et al., 2011; Niphakis et al., 2013).

### Inhibitory Evaluation of Compounds

**FIGURE 6:**
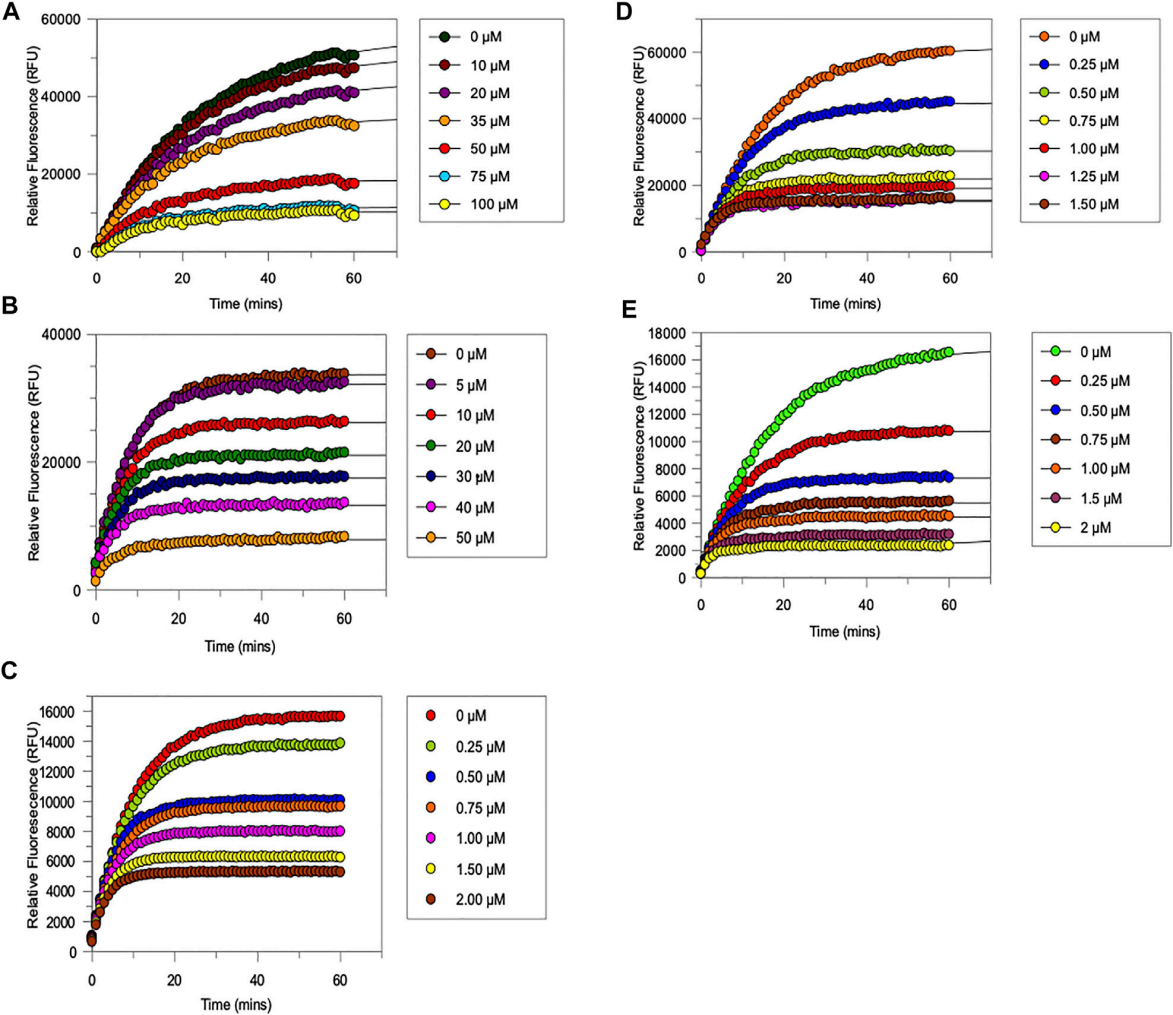
Progress curves for the trypsin-catalysed hydrolysis of Cbz-Gly-Gly-Arg-AMC in the presence of increasing amounts of **(A)** NAP851, **(B)** NAP966 **(C)** NAP897, **(D)** NAP884 and **(E)** NAP858. All assays were carried out in PBS, pH 7.4, at 37°C, using a fixed amount of trypsin (0.004 mg per well) in the presence of varying concentration of NAP851 (10–100 μM), NAP966 (5–50 μM), NAP897 (0.25–2 μM), NAP884 (0.25–1.5 μM) and NAP858 (0.25–2 μM), in the presence of a fixed concentration (50 μM) of the substrate Cbz-Gly-Gly-Arg-AMC.

**FIGURE 7:**
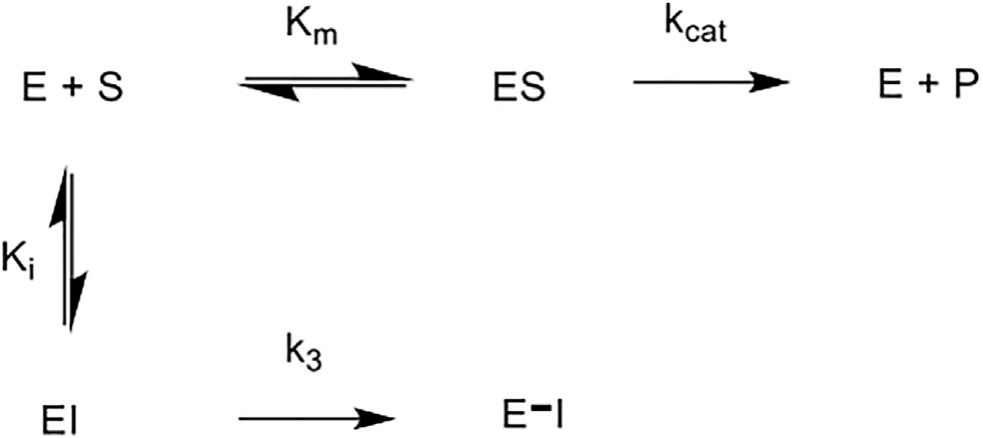
Scheme for irreversible inhibition. E: Enzyme, S: Substrate, ES: Enzyme-Substrate constant, P: Product, EI: Enzyme-Inhibitor complex, K_m_: Michaelis-Menten constant, K_i_: Inhibition constant, k_cat_: catalytic constant, k_3_: first order rate constant, E–I: irreversible enzyme-inhibitor complex.

The five novel peptides were evaluated for their ability to inhibit trypsin, in the presence of competing substrate; the results of these studies are shown in Figure 6 (A-E). These “product progress” curves are indicative of the action of irreversible inhibitors operating through the kinetic scheme illustrated in Figure 7. In this scheme, formation of the reversible enzyme-inhibitor complex (EI) is characterised by the inhibitor constant K_i_. This is converted into the covalent/irreversible complex (E-I) characterised by a first-order rate constant k_3_. The overall second-order rate constant for the inactivation of E by I is given by the ratio k_3_/K_i_ (Walker and Elmore, 1984; Tsou, 1988).

It can be appreciated from Figure 6 that each compound exhibits the characteristic active-site saturation, indicative of irreversible inhibition of trypsin. Table 2 lists the kinetic constants (K_i_, k_3_ and k_3_/K_i_) obtained for each inhibitor against trypsin. Also included in this table are the corresponding kinetic constants obtained previously for the inhibition of trypsin by the α-aminoalkyl diphenyl-phosphonate arginine analogue, QUB-TL1, that functions as an effective AP for the TLPs (Reihill et al., 2016).

**TABLE 2:** Kinetic constants determined for the inhibition of trypsin with NArg inhibitors.

Inhibitor	K_i_ (M)	k_3_ (min^−1^)	k_3_/K_i_ (M^−1^ min^−1^)^a^
*NAP851*	7.56 (±3.26) x 10^–6^	0.160 (±0.020)	2.46 (±0.858) x 10^4^
*NAP966*	2.12 (±0.31) x 10^–6^	0.401 (±0.007)	1.92 (±0.250) x 10^5^
*NAP897*	6.63 (±4.11) x 10^–7^	0.741 (±0.225)	1.35 (±0.376) x 10^6^
*NAP884*	4.20 (±1.11) x 10^–7^	0.528 (±0.064)	1.34 (±0.351) x 10^6^
*NAP858*	2.26 (±1.29) x 10^–7^	0.473 (±0.101)	2.06 (±0.884) x 10^6^
*QUB-TL1* *^b^*	1.74 × 10^–7^	0.284	1.64 × 10^6^

a Kinetic values are shown ±S.E.M., for three separate determinations.

b Kinetic values are taken from reference [30].

All five newly synthesised compounds exhibited excellent inhibitory activity against trypsin (Figure 6), which immediately confirmed our hypothesis that the use of reversed *N*-alkyl glycine residues at the P1 position would enable potent inhibition of this serine protease. In terms of inhibitory potency, the pegylated carbamate NAP858 displayed the highest 2^nd^ order rate constant (k_3_/K_i_ ≈ 2.1 × 10^6^ M^−1^ min^−1^) of all the newly synthesised compounds tested. Indeed, NAP858 is marginally more efficient than the diphenyl-phosphonate inhibitor QUB-TL1, previously reported by our group as an effective irreversible inhibitor of trypsin (Reihill et al., 2016).

With the exception of compounds NAP851 and NAP966, which exhibit low micro molar K_i_ values (≈7.6 μM and ≈2.1 μM, respectively), the remaining three compounds tested all exhibited sub-micromolar K_i_ values against trypsin.

NAP851, the only compound tested containing a triazole urea as an electrophilic leaving group, displayed the lowest inhibitory effect on trypsin out of the five inhibitors investigated, with a determined 2^nd^ order rate constant (k_3_/K_i_) of ≈2.5 × 10^4^ M^−1^ min^−1^, some 6-fold, 4-fold and 8-fold smaller than those obtained for carbamate analogues NAP966, NAP897/NAP884 and NAP858, respectively (Table 2). Additionally, it can be appreciated from the progress curves presented in Figure 6A that there continued to be a slight increase in fluorescence, due to residual substrate turnover, even after 60 min incubation of trypsin with NAP851. In contrast, all the remaining four peptides tested exhibited the ability to completely abolish substrate turnover, at prolonged incubation times (Figures 6B–E). It is interesting to note that the value of k_3_ determined for NAP851 (≈0.16 min^−1^) is the smallest of all the 1^st^-order rate constants obtained for the formation of the presumed “irreversible” E-I complex between trypsin and any of the compounds tested.

This initial study suggests the *N*-alkyl glycine arginine NHS carbamate to provide a better electrophilic moiety for targeting trypsin than does the corresponding triazole urea.

### Evaluation of “Off-Target” Inhibitory Effects

**FIGURE 8:**
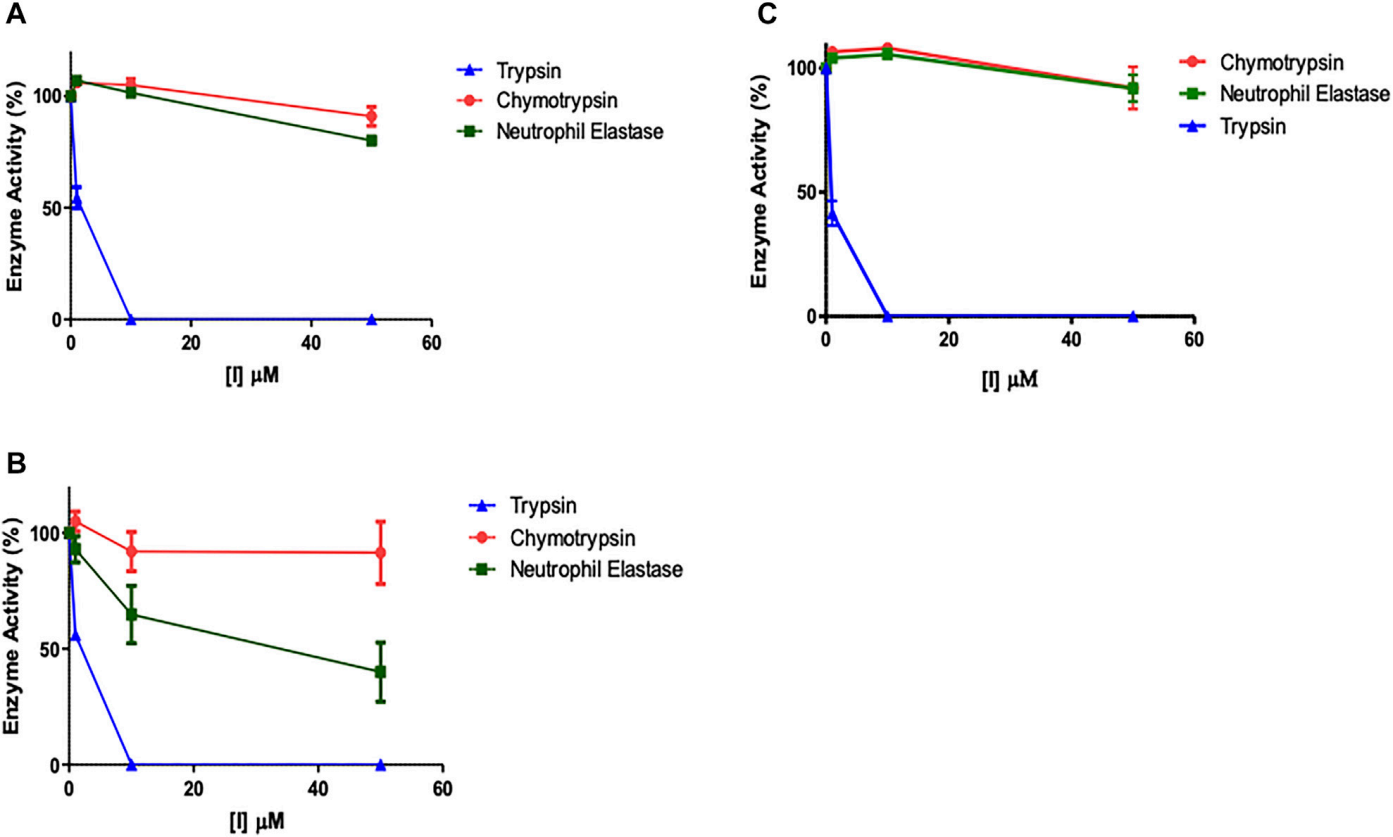
Assessment of potential “off-target” inhibitory effects of NArg analogues versus a selection of serine proteases. The impact of **(A)** NAP897, **(B)** NAP884 and **(C)** NAP858 activity on trypsin, chymotrypsin and neutrophil elastase was assessed using the cognate fluorogenic substrates. Results shown as mean ± SD (*n* = 2).

It was assumed that the presence of the NArg residue in the series of peptides synthesised in the present study would imbue them with a selective inhibitory profile for trypsin over the closely related serine proteases, elastase and chymotrypsin. In order to formally test this assumption, the potential “off-target” inhibitory effects of the lead compounds were assessed (Figures 8A–C). Given that NAP851 and NAP966 were the least effective inhibitors of trypsin tested and that the former exhibited incomplete quenching/inhibition of substrate hydrolysis catalysed by trypsin, neither of these two peptides were included in these “off-target” studies.

It is clear from Figure 8 that NHS carbamates NAP897 (Figure 8A), NAP884 (Figure 8B) and NAP858 (Figure 8C) exhibit a pronounced selectivity of action in inhibiting trypsin across the range of concentrations studied. In particular, peptides NAP897 and NAP858 exhibit only very slight inhibition against both chymotrypsin and NE “off-targets” (<10% relative to controls) and only at the highest concentration of each that was tested (50 μM). In contrast, although the peptide NAP884 exhibited similarly discriminating inhibitory behaviour against trypsin compared to chymotrypsin, substantial “off-target” inhibition against NE was observed, causing an approximate 50% decrease in activity, when tested at 50 μM (Figure 8B).

### Evaluation of Compounds NAP858, NAP884 and NAP897 as Activity Probes (APs)

**FIGURE 9:**
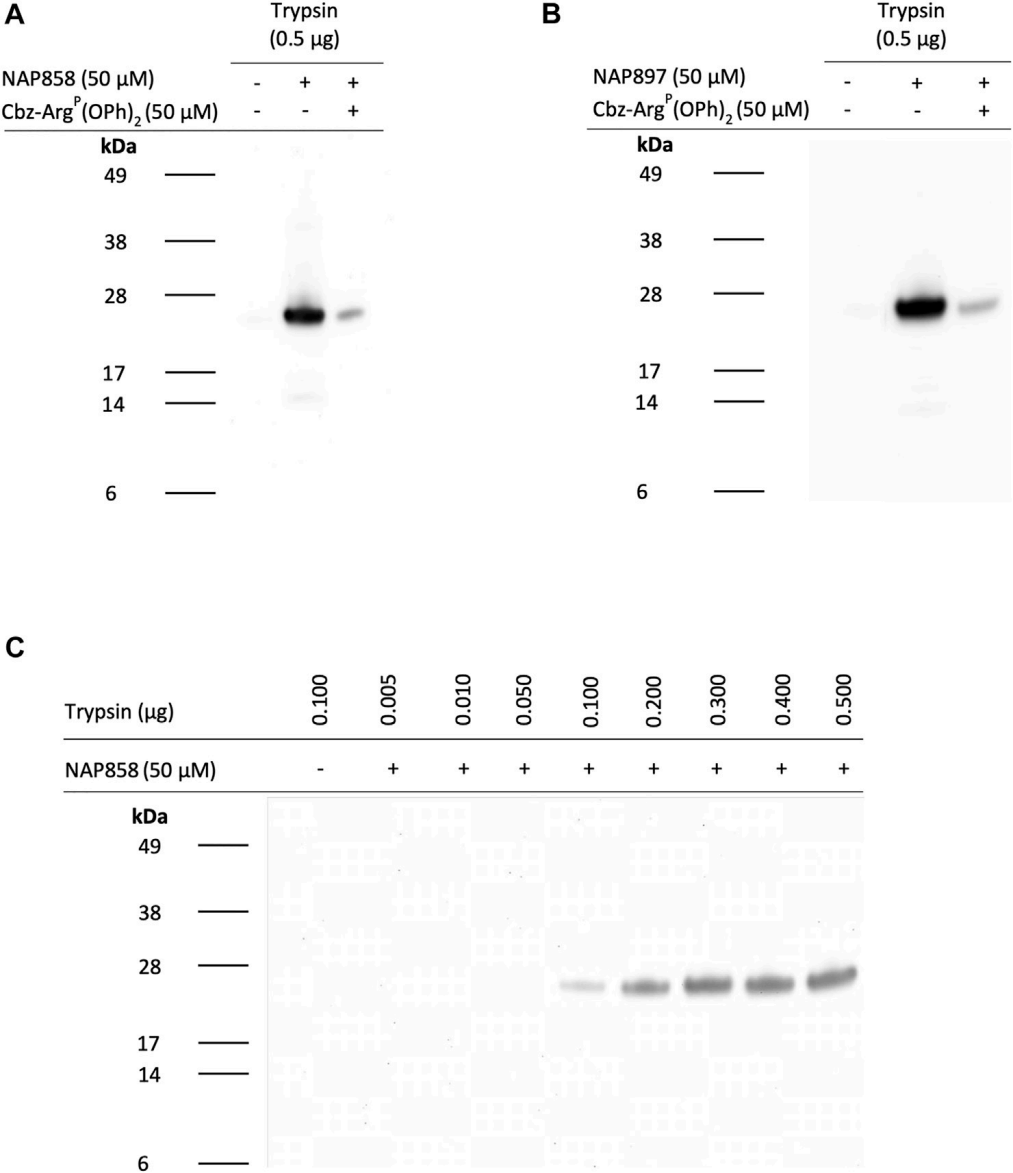
NAP858 and NAP897 function as activity-based probes (ABPs) for trypsin. Samples of trypsin were treated with either **(A)** NAP858 (+) or **(B)** NAP897 (+), used at a concentration of 50 μM, for 30 min, at 37°C. For each blot a vehicle control sample was included (−/−) as well as a further sample of trypsin pre-treated with Cbz-Arg^P^(OPh)_2_ (+) prior to incubation with the APs. **(C)** Solutions containing ascending amounts of trypsin (0.01–0.5 μg/ml) were also incubated with NAP858 (+), under exactly the same conditions as indicated for panels **(A)** and **(B)**, above. All treated samples were reduced prior to SDS-PAGE. Western blotting analysis was then performed which allowed visualisation of the biotinylated inhibitor-protease complex using streptavidin-HRP.

We next evaluated the potential of NAP858, NAP884 and NAP897 to function as APs, firstly, for trypsin and then for a range of TLPs. Figure 9 illustrates the ability of the biotinylated NHS carbamates NAP858 and NAP897 (Figure 9A and Figure 9B, respectively) to disclose the presence of trypsin, in samples that were incubated with either inhibitor (used at a final concentration of 50 μM), prior to SDS-PAGE protein resolution, Western blotting and detection using a streptavidin-HRP conjugate. Treatment of the samples containing trypsin with NAP858 or NAP897 resulted in the labelling of a single protein band corresponding to the correct molecular mass of trypsin (≈23 kDa). Furthermore, pre-treatment of duplicate samples with Cbz-Arg^P^(OPh)_2_, a previously reported active site directed inhibitor of trypsin (Martin and Walker, 2017), prior to incubation with NAP858 or NAP897, caused a substantial reduction in the intensity of this 23 kDa band, further supporting the kinetic data reported above that these NHS carbamates are active site directed irreversible inhibitors.

Further investigation was then carried out to determine the limit of detection for trypsin, using NAP858 as an AP (Figure 9C). Under the experimental conditions described, as little as 100 ng of trypsin was visualised using NAP858, with excellent visual detection enabled from 200 ng and above. Similar results were obtained using NAP897 as an AP (data not shown).

**FIGURE 10:**
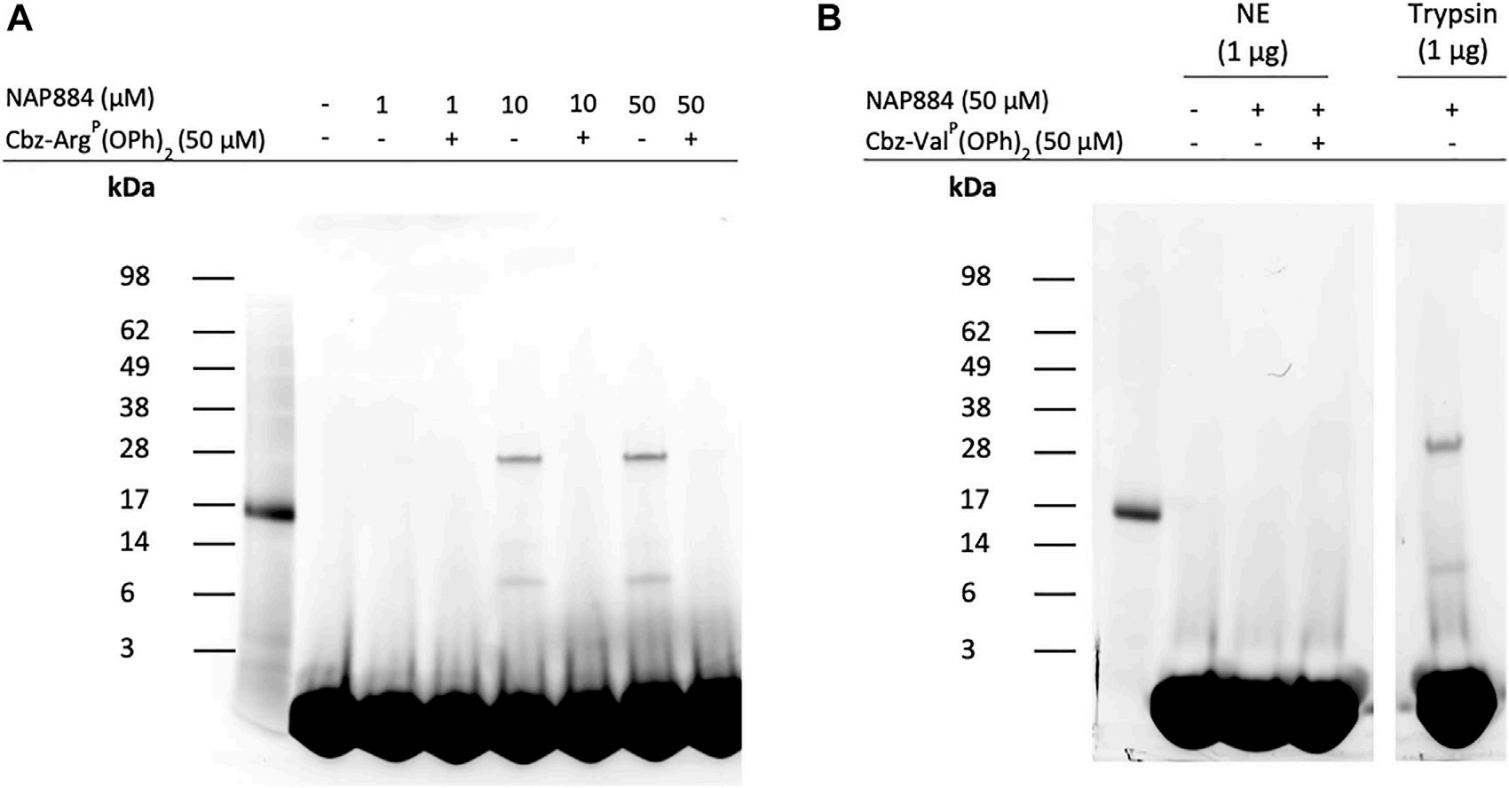
NAP884 functions as an AP for trypsin **(A)** Samples of trypsin (1 μg), were treated with varying concentrations of NAP884 (±Cbz-Arg^P^(OPh)_2_), for 30 min, at 37°C. DMSO (final conc. 25% v/v), rhodamine-azide (final conc. 60 μM), copper (II)-TBTA complex (final conc. 1 mM) and freshly prepared sodium ascorbate (final conc. 1 mM) were then added. The samples were vortexed briefly and gently agitated in the dark, 1 h, at room temperature. They were then denatured and subjected to SDS-PAGE **(B)** NE or trypsin (1 μg) were treated with NAP884 (±Cbz-Val^P^(OPh)_2_) for 30 min, at 37°C and processed as above.

We also investigated the use of the “click chemistry” compatible peptide NAP884 as an AP for trypsin. Figure 10A shows the effect of incubating trypsin samples with varying concentrations of NAP884 followed by a click chemistry reaction with rhodamine-azide. The labelled proteins were then resolved by SDS-PAGE and detected by UV illumination. NAP884, when used at concentrations of 10 and 50 μM, enabled the detection of trypsin as a fluorescent band corresponding to the correct mass of 23 kDa. Trypsin was however not detectable using NAP884 at a concentration of 1 μM possibly due to substantial inhibition of trypsin as evidenced by the product progress curves recorded in Figure 6D, which may have led to reduced rates for the subsequent “click chemistry” reaction. Additional bands of apparent lower molecular mass were also detected, suggesting the presence of some cleaved trypsin fragments containing the labelled active site. Once again, pre-treatment of trypsin with Cbz-Arg^P^(OPh)_2_ prior to exposure to NAP884, abrogated any labelling/detection of trypsin, providing additional evidence for the active site-directed action of the NHS carbamate inhibitors.

As discussed above, NAP884 displayed some unexpected inhibition of NE, according to the “off-target” inhibition studies employing fluorometric substrate inhibition assays which could impact on the usefulness of NAP884 as a selective AP for trypsin and TLPs. It was therefore assessed whether NAP844 could disclose/detect NE in samples containing this protease. The results of these ABP experiments are shown in Figure 10B and clearly demonstrates the detection/labelling of the ‘trypsin control’ and the complete absence of labelling of NE, by NAP884. This result appears to be at variance with the “off-target” inhibition studies described above. However, this apparent contradiction can be explained if NAP884 functions only as a competitive reversible inhibitor of NE, meaning the inhibitory effect would then be reversed after subjecting the samples of NE treated with NAP884 to SDS-PAGE. Consequently, the evidence suggests that NAP884 still has potential as a selective AP for trypsin.

**FIGURE 11:**
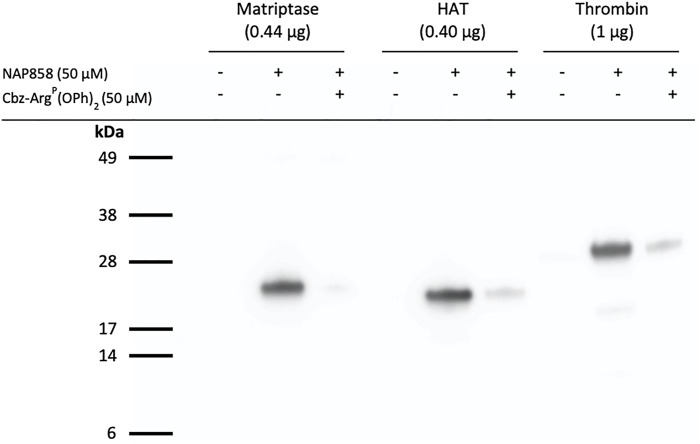
NAP858 functions as an activity probe (AP) for TLPs. Proteases, matriptase, HAT and thrombin, were treated (±) NAP858 (±Cbz-Arg^P^(OPh)_2_), for 30 min at 37°C. Samples were resolved by SDS-PAGE under reducing conditions with streptavidin-HRP used to visualise the inhibitor-protease complex.

We have previously demonstrated that QUB-TL1, a diphenyl phosphonate AP is a potent inhibitor of matriptase and HAT (Reihill et al., 2016). Given that this present study demonstrated that the biotinylated and pegylated *N*-alkyl glycine NHS carbamate, NAP858 was more effective at inhibiting trypsin than QUB-TL1, we decided to investigate the ability of this NAP858 to function as an AP. Figure 11 shows that NAP858 does indeed function as an AP for both proteases, as evidenced by the detection of bands corresponding to the correct molecular weight of matriptase (26 kDa) and HAT (25 kDa). Additionally, Figure 11 demonstrates that NAP858 can act as an AP for thrombin, a TLP of the clotting system (Davie et al., 1991; Bendel et al., 2011), as evidenced by the labelling of the thrombin heavy (catalytic) chain (31 kDa), establishing its potential as a broad-spectrum AP. Finally, pre-treatment of samples of each of these three TLPs with Cbz-Arg^P^(OPh)_2_, prior to incubation with NAP858, resulted in the substantial reduction of detectable signal for each protease, reinforcing our contention that the labelling is active-site directed.

The positive outcomes to these studies provided the necessary impetus for us to conduct ABP on biological samples, using NAP858. Since TLPs, such as Per a 10, have previously been reported in German cockroach *Blattella germanica* extracts (Arizmendi et al., 2011), we decided to initially investigate the effectiveness of NAP858 as an AP, in this context. In addition, since TLPs have also been isolated from the American cockroach *Periplaneta americana*, we also performed ABP studies on commercially available extracts of this major asthma allergen (Sudha et al., 2008).

**FIGURE 12:**
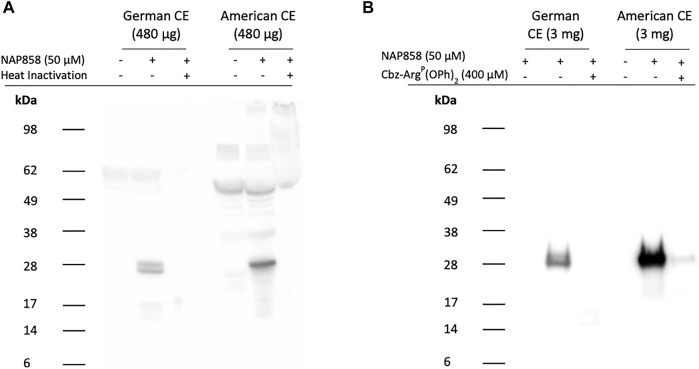
ABP of cockroach extracts for trypsin-like proteases using NAP858 **(A)** Cockroach extract (480 μg) was treated (±) with NAP858 (± heat inactivation at 95°C, for 10 min), for 30 min, at 37°C or **(B)** cockroach extract (3 mg) was pretreated with neutroavidin agarose beads to remove endogenous biotinylated proteins and the supernatant probed (±) with NAP858 (±Cbz-Arg^P^(OPh)_2_), for 30 min, at 37°C. Purification of the labelled proteins was achieved by incubation with a fresh batch of washed neutravidin agarose beads. Bound proteins were extracted from beads using SDS-containing reducing treatment buffer. In both cases **(A,B)** samples were subsequently labelled and visualised using streptavidin-HRP.

ABP studies of American cockroach extract probed with NAP858 are presented in Figure 12A and show the detection of a single, intense band with an apparent molecular mass of 25 kDa. This corresponds to the molecular mass reported for the serine protease Per a 10, previously isolated from this cockroach species (Sudha et al., 2008). ABP of *B*. *germanica* extracts with NAP858 revealed a close doublet of protein bands of similar molecular mass (≈25 kDa) to both trypsin and Per a 10. It is also apparent from this figure that there is substantial “background noise” in the “no probe” controls, due, most probably, to the presence of endogenously biotinylated proteins in the extracts. In order to produce cleaner ABP blots, NAP858 was used as an affinity purification handle to enrich/purify the TLPs present in the German and American cockroach extracts. It can be appreciated that this procedure resulted in a significant reduction in background noise and enhanced detection of the NAP858-labelled TLPs (Figure 12B).

### Covalent Docking of NAP858

**FIGURE 13:**
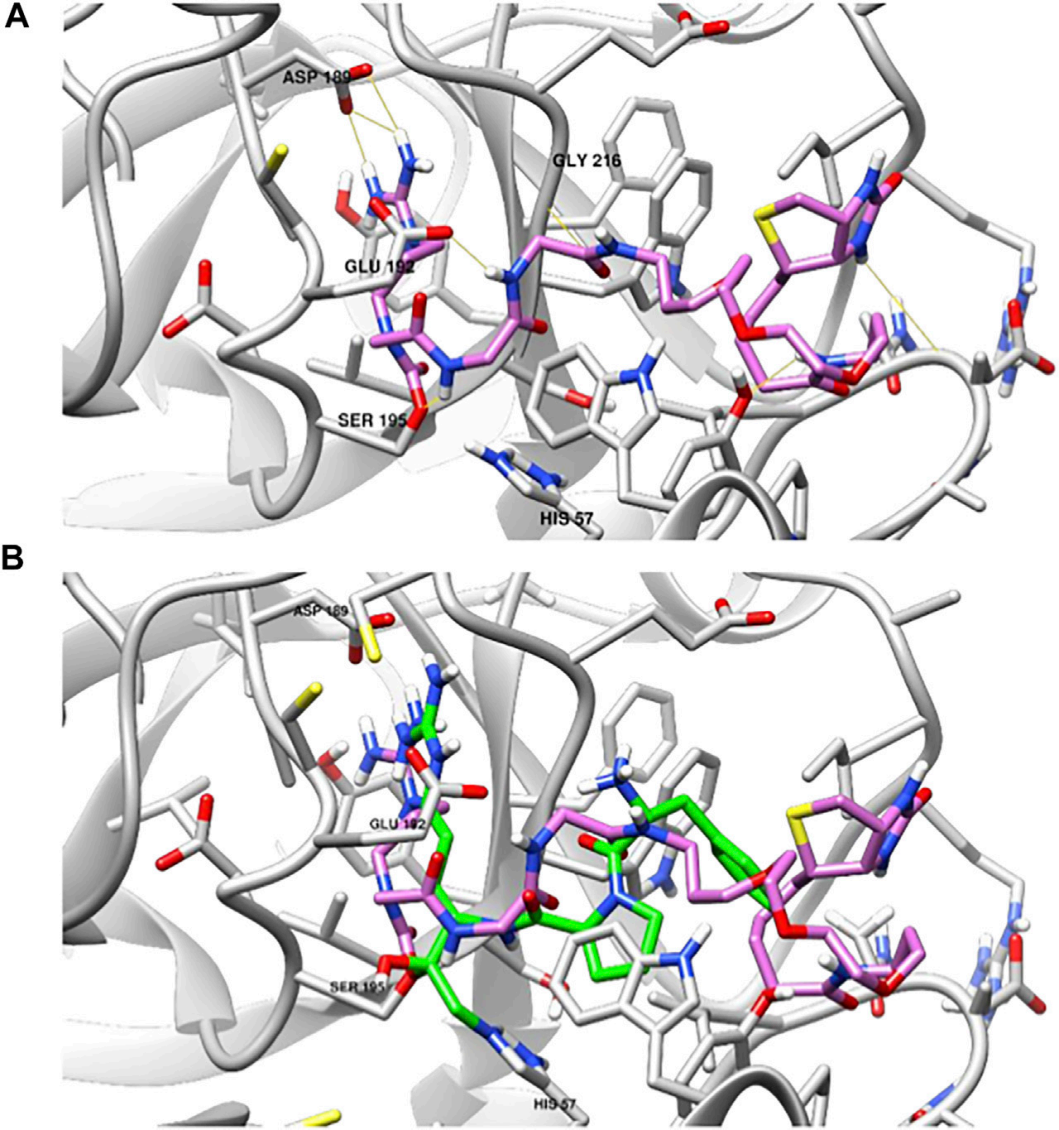
Covalent docking studies of NAP858 with thrombin. **(A)** Active site of thrombin (PDB: 1DWE) covalently bound to NAP858 *via* carbamate linker to SER195 (hydrogen bonds indicated in yellow). **(B)** Superimposed view of NAP858 (pink) and *H*-D-Phe-Pro-Arg-CMK (green), covalently bound to the active site of thrombin.

To provide further understanding of the binding of these compounds to the active site of TLPs, we performed a covalent docking study of NAP855 to the active site of thrombin as an example TLP. We have shown that NAP858 acts as an irreversible inhibitor of thrombin (Figure 11), with the ability to label the active site of the thrombin heavy chain in a western blot. We hypothesised that NAP858 would bind to the active site in a reversed manner (C⇨N) to normal peptide substrates, with the NArg group acting as the P1 residue and a carbamate linkage formed between NAP858 and the active site serine residue (SER195) (Figure 5). The lowest energy binding pose (Figure 13A) illustrates that NAP858 adopts a pose whereby the basic NArg side-chain docks into the S1 pocket of thrombin forming a salt-bridge with the acidic aspartic acid residue (ASP189) at the base of the S1 pocket. This aligns with the binding of the irreversible chloromethylketone (CMK) inhibitor *H*-D-Phe-Pro-Arg-CMK co-crystallised with thrombin (Figure 13B), with the both the P1 arginine residue in the chloromethylketone, and the P1 NArg residue in NAP858, filling the S1 binding pocket of thrombin. Furthermore, despite the reversed sequence (C⇨N), the inhibitor backbone is still able to form hydrogen bonds to the protease binding pocket (e.g. GLY216).

## Discussion

We hypothesised that peptides containing an *N*-alkyl glycine mimetic of a P1 arginine residue (NArg) and functionalised with a carbamate or triazole-urea linker group, of the types previously reported to inhibit serine hydrolases (Adibekian et al., 2011; Chang et al., 2013; Niphakis et al., 2013; Cognetta et al., 2015; Chen et al., 2019; Otrubova et al., 2019), would furnish potential inhibitors of trypsin and TLPs. This reasoning was based on the fact that topographically similar and previously reported P1 aza-amino acid-derived carbamates function as inhibitors of this protease family (Powers and Gupton, 1977; Nardini et al., 1996). The generalised chemical structure of the NArg containing peptides synthesised in the present study, along with those for an aza-arginine containing analogue and a standard peptide substrate, are shown in Figure 4. It was postulated that the NArg peptides would dock into the active site in a reversed manner (C⇨N) to that of the aza-peptides and standard peptide substrates (N⇨C) with the side-chain of the P1 NArg residue being expected to dock into the S1 pocket of trypsin-like proteases with the P2 and P3 glycine residues, which are common features of the series of peptides synthesised in the present study, projecting into the S2 and S3 binding pockets (“P” and “S” nomenclature according to Schechter and Berger, 1967). The guanidine function of the P1 NArg residue would be expected to interact with the acidic aspartic acid residue located at the base of the S1 pocket in this protease family, in a similar manner to normal trypsin substrates (Graf et al., 1988). Although the orientation of the amide bonds within the NArg peptide sequences are reversed, the carbonyl function of each are in an identical position to that of a standard peptide substrate and aza-peptide. Therefore, it was anticipated that hydrogen bonding between the NArg containing peptides and their target trypsin-like serine protease would be maintained.

Although this “reverse binding” would be unusual, it is not without precedent. For example, it has been established that the *N*-terminal tetrapeptide sequence of the leech derived peptide hirudin, an exceptionally potent and specific inhibitor of thrombin, binds in a retro manner to a normal substrate (Grutter et al., 1990). This retro binding is also recapitulated with the synthetic, truncated hirudin mimetic, hirunorm (De Simone et al., 1998). Intriguingly, anophelin, another thrombin-directed inhibitor isolated from Anopheles mosquitoes, also displays this reverse binding mode (Figueiredo et al., 2012). Additionally, it has been established that particular Fab and scFv antibodies derived from phage display libraries, function as potent inhibitors of the type II transmembrane serine protease matriptase, through insertion of one of their exposed surface loops, in a reverse orientation, into the substrate-binding pocket of this trypsin-like serine protease (Schneider et al., 2012).

This hypothesis of reversed binding was supported by the findings from the covalent docking study of NAP858 into thrombin (Figure 13), which indicated the correct location of the P1 NArg residue interacting with the S1 aspartic acid residue. Despite the reversed orientation of the amide bonds, hydrogen bonding to the protease backbone by the carbonyl groups in NAP858 was still possible. The binding of NAP858 to thrombin also aligns with the co-crystallised structure of *H*-D-Phe-Pro-Arg-CMK covalently bound to the thrombin active site. This would also suggest that further modification to the structure of NAP858 at the -Gly-Gly- (-P2-P3-) residues should enable improved binding to the S2 and S3 binding pockets, allowing conversion of the broad-spectrum AP into one with more specific activity against a target TLP.

We postulated that the NArg-containing peptides would function as irreversible inhibitors of trypsin and TLPs via a mechanism, similar to that established for aza-peptide carbazates (Powers and Gupton, 1977; Nardini et al., 1996). Figure 5 summarises the postulated mechanism. It is proposed that the NArg-containing peptides dock into the active site of the protease, with the side-chain on the NArg residue occupying the S1 subsite. Nucleophilic attack by the serine hydroxyl group on the carbonyl function of the NArg carbamate or triazole urea functionality would be expected to occur as reported for the aza peptide inhibitors (Powers and Gupton, 1977; Nardini et al., 1996), leading to the expulsion of the leaving group. By analogy with aza peptides, the inductive effect of the nitrogen atom of the NArg residue in the alpha position to the carbamate carbonyl, reduces the electrophilicity of the latter, resulting in the formation of a stable acyl-enzyme intermediate, which is hydrolysed much more slowly than that formed during normal substrate cleavage, effectively yielding irreversible inhibition of the protease.

While we have yet to formally confirm this mechanism, by direct observation of the formation of this postulated acyl enzyme intermediate, the kinetic analysis of the inhibition processes and the ability to detect trypsin and various TLPs, inactivated by the NArg-containing peptides, following SDS-PAGE resolution, lends credence to this proposed inhibitory mechanism. This notwithstanding, we have provided solid evidence of the potential utility of peptide NArg (alkyl glycine) carbamates as broad-spectrum activity probes for the TLPs, and given their relative ease of synthesis, we anticipate that they will provide an alternative to phosphonate (Abuelyaman et al., 1997; Reihill et al., 2016; Lovell et al., 2021), phosphinate (Kahler and Verhelst, 2021) and fluorophosphonate (Liu et al., 1999; Kidd et al., 2001) inhibitors for activity-based profiling applications, due to the synthetically more challenging methods required for the preparation of the latter three compound classes. Although formal stability studies in human serum will provide further confidence for more wide-ranging biological applications, we have demonstrated the value of these compounds for both detecting and purifying proteases from complex biological samples, with successful labelling of TLPs in cockroach extracts (Figure 12). This would suggest sufficient levels of both stability and specificity of these compounds in the presence of a variety of other proteins and enzymes. This is not always the case for commonly used protease labelling tools such as chloromethylketones, which are known to have poor stability in biological matrices (Powers et al., 2002) or fluorophosphonates which will label all serine hydrolase enzymes indiscriminately (Liu et al., 1999; Kidd et al., 2001). Therefore, these compounds have a high potential value as new APs for both the detection and purification of proteases in extremely complex biological samples and are a useful addition to the available tools for ABP applications.

## Data Availability

The raw data supporting the conclusion of this article will be made available by the authors, without undue reservation.
