# Supplementary material for: Novel Inhibitors and Activity-Based Probes Targeting Trypsin-Like Serine Proteases
Source: Front Chem. 2022 Apr 21;10:782608. doi: 10.3389/fchem.2022.782608 (PMC9068901; doi:10.3389/fchem.2022.782608)
Supplement: Supplementary file 1 [file DataSheet1.PDF]

Novel inhibitors and activity-based probes targeting trypsin-like serine proteases

Timothy E. G. Ferguson[1]*, James A. Reihill[1], S. Lorraine Martin[1], Brian Walker[1]

[1]Biomolecular Sciences Research Group, School of Pharmacy, Queen's University Belfast, Northern Ireland, United Kingdom

Supplementary Information

Supplemental Methods:

1. Synthesis of NArg Inhibitors (related to Figure 1)

Nα-Fmoc-Gly-Gly-Ahx-Lys(Biotin)-Wang resin

Fmoc-Lys(Mtt)-Wang resin (Bachem) (833 mg, 0.5 mmol) was swollen in DCM for 30 minutes. The lysine side-chain ε-amine was deprotected by treatment with TFA/TIPS/DCM (5:5:90) (2 x 15 mins), after which time treatment of a resin sample with neat TFA indicated no Mtt protected amine remained and Kaiser analysis indicated presence of deprotected amine. The resin was washed with DCM, 1% (v/v) DIPEA in DMF, DMF and DCM. Biotin was then coupled to the side chain amine overnight by addition of D-Biotin (488.6 mg, 2 mmol), HOBt (306.4 mg, 2 mmol), DIC (309 μl, 2mmol) and DIPEA (348 μl, 2 mmol) in NMP, until complete coupling was achieved. The peptide was then elongated using standard methods of Fmoc deprotection and DIC/HOBt coupling. Following final coupling of the second Fmoc- Gly-OH residue, the peptide-resin was washed thoroughly with DCM and dried overnight in vacuo. Following analysis of the resin using the protocol for Fmoc determination, the loading of the peptide-resin was determined to be 0.41 ± 0.02 mmol/g.

Detailed synthesis of Triazole urea-Arg-Gly-Gly-Ahx-Lys(Biotin)-OH (NAP851)

H-NArg-Gly-Gly-Ahx-Lys(Biotin)-Wang resin was synthesised on a 0.05 mmol scale as described previously for NAP897, and dried in vacuo (2 hours). The dried resin was suspended in anhydrous DCM (10 ml), followed by addition of 2,6-lutidine (3 ml) and triphosgene (237.4 mg, 0.8 mmol) (Warning: Risk of phosgene gas production. Triphosgene should be handled in an efficient fume-hood. All contaminated glassware should be rinsed with NaOH solution). The resin was agitated gently for 15 minutes after which time chloranil analysis indicated absence of secondary amine. The resin was filtered and washed with anhydrous DCM, followed by addition of a solution containing 1,2,4-triazole (207.2 mg, 3 mmol), DMAP (1.2 mg, 0.01 mmol) and 2,6-lutidine (1 ml) in anhydrous DMF and agitated overnight. The resin was filtered and a repeat solution was added for a further 6 hours. The resin was then washed and dried overnight in vacuo followed by cleavage as previously described to give title compound as a brown oil (116.4 mg, quantitative yield). ESI-MS m/z 852.39 [M + H^+], 756.42 (-$COC_2H_2N_3$) [M + H^+] (calculated $C_{35}H_{58}N_{14}O_9S$: 851.00).

Detailed synthesis of PfpOCO-NArg-Gly-Gly-Ahx-Lys(Biotin)-OH (NAP966)

This peptide was synthesised in a similar manner to NAP851 on a 0.08 mmol scale, with the exception that following reaction of the NArg resin with triphosgene a solution of pentafluorophenol (27.6 mg, 0.15 mmol), 2,6-lutidine (0.5 ml) and DMAP (1.2 mg, 0.01 mmol) in anhydrous DMF was added for 3 hours. This was followed by standard washing, drying and cleavage protocols to give the desired compound (107.5 mg). ESI-MS m/z 756.42 (-COOPfp) [M + H^+], 966.39 [M+ H^+] (calculated $C_{39}H_{56}F_5N_{11}O_{10}S$: 966.0).

Detailed synthesis of SuOCO-NArg-Gly-Gly-Ahx-Lys(Biotin)-OH (NAP897)

Fmoc-Gly-Gly-Ahx-Lys(Biotin)-Wang resin (244 mg, 0.1 mmol) was swollen and deprotected using standard protocols. To the washed resin was added a solution containing

Fmoc-N-[3- (N'-Pbf-guanidino)-propyl]-glycine (PolyPeptide Laboratories) (194.6 mg, 0.3 mmol), HOBt (45.96 mg, 0.3 mmol) and DIC (46.5 µl, 0.3 mmol) in NMP, followed by addition of DIPEA (52.3 µl, 0.3 mmol). Upon completion of the coupling reaction, following overnight agitation, the resin was washed and deprotected as previously described. The resin was suspended in anhydrous DCM/DMF (1:1), to which was added N,N'-disuccinimidyl carbonate (DSC) (76.9 mg, 0.3 mmol) and 2,6-lutidine (35 µl, 0.3 mmol). The suspension was agitated for 6 hours until complete reaction had occurred as indicated by a negative chloranil result. The resin was washed with anhydrous DMF (x 1) and DCM (x 3) and dried overnight in vacuum. Cleavage of the desired peptide was carried out with addition of TFA/TIPs/DCM (95:2.5:2.5, 5 ml) for two hours, followed by filtration and concentration under reduced pressure. The peptide was precipitated in diethyl ether, collected by centrifugation and washed with further diethyl ether, before being dried in vacuo to give the title compound as an off-white solid of the TFA salt (140 mg, quantitative yield). ESI-MS m/z 378.71 (-COOSu) [2M + H+]/2, 756.42 (-COOSu) [M + H+], 773.43 (-COOSu) [M+ NH3+], 897.43 [M + H+] (calculated $C_{37}H_{60}N_{12}O_{12}S$: 897.02).

Detailed synthesis of SuOCO-NArg-Gly-Gly-Ahx-Lys-(Epb)-OH (NAP884)

Synthesis was carried out on a 0.1 mmol scale in an identical manner to NAP897, with the exception that instead of coupling biotin to Mtt-deprotected Fmoc-Lys-Wang resin, 4-(3-ethynyl-phenylcarbamoyl)-butyric acid was used. Following standard coupling and cleavage methods the title compound was acquired (37.3 mg, 37%). ESI-MS m/z 884.43 [M+ H+] (calculated $C_{40}H_{57}N_{11}O_{12}$: 883.96).

Synthesis of the Biotin-PEG NHS Carbamate Probe (related to Figure 2)

Detailed synthesis of SuOCO-NArg-Gly-Gly-PEG-Biotin (NAP858)

Synthesis of this peptide was carried out using Biotin-PEG NovaTag resin (Novabiochem) (104 mg, 0.05 mmol). The required sequence was then synthesised using standard protocols similar to the procedure described for NAP897, to give the desired compound as a colourless oil (89.5 mg). ESI-MS m/z 359.21 (-COOSu) [M + 2H+]/2, 717.41 (-COOSu) [M+ H+], 858.42 [M+ H+] (calculated $C_{35}H_{59}N_{11}O_{12}S$: 857.98).

Spectra for NAP858

Following successful synthesis, the compounds were analysed by MS (Table 1) and HPLC prior to investigation of their inhibitory properties. The ESI-MS spectrum is shown below for the lead compound NAP858. As experienced previously within our laboratory for the class of compound containing an *N*-alkyl glycine NHS carbonate, the MS showed the expected molecular weight as well as the presence of a compound with loss of the reactive NHS carbamate warhead. HPLC analysis showed higher purity with the phenylalanine and valine analogues within the library, than the lysine and arginine analogues such as NAP868, suggesting the basic side chain of these residues contributed to the loss of the NHS carbamate as it passed down the HPLC column. This is unsurprising as this observation is also made with diphenyl phosphonate analogues, where basic conditions especially in the presence of alcohols can result in a loss of the phenol esters.

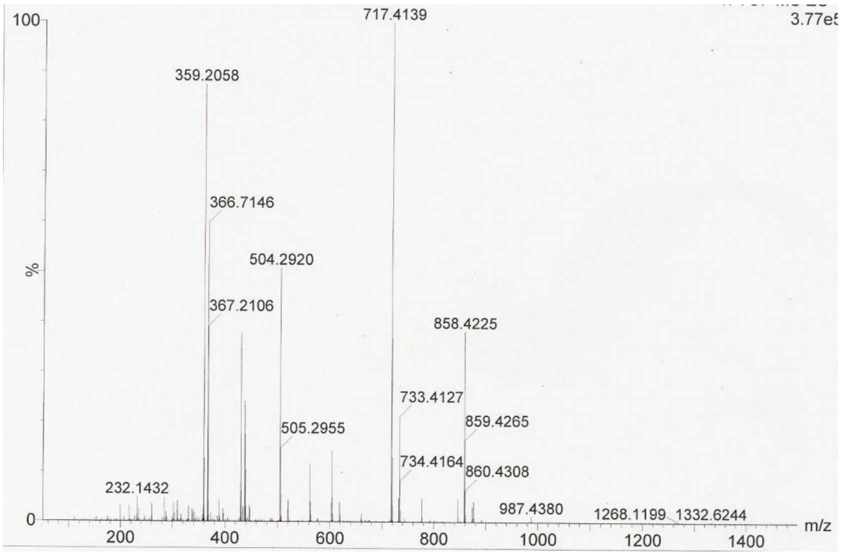

Supplementary Figure S1: ESI-MS Spectrum for NAP858.

m/z 359.21 (-COOSu) $[M + 2H^{+}]/2$, 717.41 (-COOSu) $[M + H^{+}]$, 858.42 $[M + H^{+}]$ (calculated $C_{35}H_{59}N_{11}O_{12}S$: 857.98).

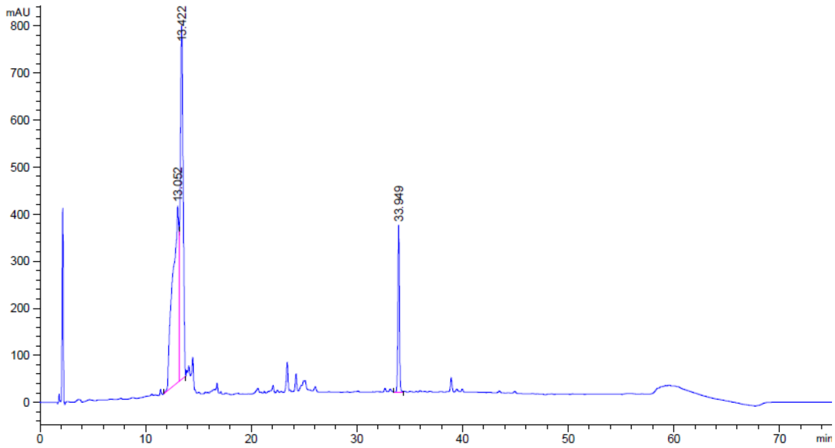

Supplementary Figure S2: HPLC Chromatogram for NAP858.
